# The complete mitochondrial genomes of the flapper skate *Dipturus intermedius* and the longnose skate *Dipturus oxyrinchus*

**DOI:** 10.1080/23802359.2022.2064248

**Published:** 2022-05-26

**Authors:** Tanja N. Schwanck, Aurelien N. Delaval, Leslie R. Noble, Peter J. Wright, David W. Donnan, Catherine S. Jones

**Affiliations:** a School of Biological Sciences, University of Aberdeen, Aberdeen, UK; b Faculty of Biosciences and Aquaculture, Nord University, Bodø, Norway; c Marine Scotland Science, Marine Laboratory, Aberdeen, UK; d NatureScot, Perth, UK

**Keywords:** *Dipturus intermedius*, *Dipturus oxyrinchus*, flapper skate, common skate, *Dipturus batis*, mitogenome

## Abstract

We describe the complete mitochondrial genomes of the flapper skate *Dipturus intermedius* (Parnell 1837) and the longnose skate *Dipturus oxyrinchus* (Linnaeus 1758), which have been obtained by Sanger sequencing. We report the length of the sequences to be 16,906 and 16,911 bp, respectively. The length and structure of gene regions, containing 13 protein-coding regions, 22 tRNA genes, two rRNA genes, and two non-coding areas, resemble those of related skate species. Despite *D. intermedius* being considered a cryptic species with *D. batis*, the full mitogenomes confirm that *D. intermedius* and *D. oxyrinchus* are more genetically similar. In comparison to other *Dipturus* species, *D. intermedius* is missing a whole codon in its cytochrome oxidase subunit 2 gene. These mitogenomes will be a useful resource furthering investigation of the population genetic differences and evolutionary history of skate species.

In the Northeast Atlantic, several skate species (family Rajidae) play an important role in fisheries. They make up more than 40% of elasmobranch landings, most from the North Sea and Celtic Sea (Ellis et al. 2008). Though the vulnerability of skate species has been widely recognized, conservation efforts are hindered by reoccurring taxonomic confusion (Dulvy et al. 2000; Iglésias et al. 2010). Recently, the common skate *Dipturus batis* was shown to be two distinct species, now called the blue skate *Dipturus batis* (previously *D. cf. flossada*) and the flapper skate *Dipturus intermedius* (previously *D. batis* and *D. cf. intermedia*) (Griffiths et al. 2010; Last et al. 2016). Phylogenetic analysis based on short mitochondrial markers showed these two species were not the most closely related taxa, instead the longnose skate *Dipturus oxyrinchus* is included in the clade as a sister species to *D. intermedius* (Iglésias et al. 2010).

To contribute to the knowledge of genetic differences between these commonly confused closely related species, we complement the already described mitogenome of the blue skate *D. batis* (Delaval et al. 2020) with full mitochondrial genomes of the flapper skate *D. intermedius* and the longnose skate *D. oxyrinchus.* Fin clips were collected of *D. intermedius*, north coast of Scotland (59° 13′ 51.6″N, 3° 29′ 49.1994″W) during a Marine Scotland Science survey under UK Home Office License, and of *D. oxyrinchus*, Norway (61° 29′ 17.052″N, 5° 16′ 23.7174″E) during a University of Bergen research cruise, which are now deposited in the Natural History Museum London (accession numbers NHMUK014943803 and NHMUK014943802, respectively, contact Jackie Mackenzie-Dodds j.mackenzie-dodds@nhm.ac.uk). No further ethical permissions were required for this study. Species identifications were based on morphological characteristics (Iglésias et al. 2010) and confirmed by genetic barcoding. Genomic DNA was extracted using a phenol–chloroform extraction method (Sambrook et al. 1989). Based on mitochondrial genomes accessible on GenBank (KF318309; KR152643; MF278961), universal primers for the genus *Dipturus* were designed in Geneious V. 11.1.5 (Kearse et al. 2012) using Primer3 V. 2.3.7 to amplify 23 overlapping fragments. PCR products were purified with the QIAquick PCR Purification Kit (Qiagen, Hilden, Germany) and Sanger sequenced (Genewiz, Essex, UK). Resulting sequences were trimmed and mapped against the mitochondrial genome of *D. kwangtungensis* as a reference (KF318309) in Geneious V. 11.1.5 (Kearse et al. 2012). Between 32 and 35 sequences were assembled for full high-quality coverage of each mitogenome and a consensus sequence generated. Regions of the mitochondrial genome were annotated with reference to available mitochondrial genomes.

The assembled products produced a consensus sequence of 16,906 bp in *D. intermedius* (MT890688) and 16,911 bp in *D. oxyrinchus* (MT890691), the mitogenomes resembling those of other *Dipturus* species in size and gene order (Vargas-Caro et al. 2016; Delaval et al. 2020). Like other *Dipturus* relatives, they contain 13 protein-coding regions, 22 tRNA genes, two rRNA genes, and two non-coding areas (control region and the origin of L-strand replication). Nucleotide frequencies in both species were very similar, with the mitogenome of *D. intermedius* consisting of 29.9% A, 26.8% C, 14.4% G, and 28.9% T (G + C composition of 41.2%) and that of *D. oxyrinchus* 29.9% A, 26.9% C, 14.5% G, and 28.8% T (G + C composition of 41.3%). *D. intermedius* has the shortest mitochondrial genome among published sequences of *Dipturus* skates, missing one codon (GAA, glutamic acid) in the cytochrome oxidase subunit 2 gene compared with other species in the genus, while all other genes are of the same length as in other skate species. Phylogenetic reconstruction based on the maximum-likelihood method by RAxML V. 8.2.11 (Stamatakis 2014) in Geneious V. 11.1.5 (Kearse et al. 2012) under the GTR + G+I substitution model with 1000 bootstrap replicates grouped *D. intermedius* and *D. oxyrinchus* as sister taxa (Figure 1), confirming previous phylogenetic analyses based on partial mitochondrial markers (Iglésias et al. 2010; Carugati et al. 2022). Correspondingly, the mitogenome of *D. intermedius* shows a slightly higher pairwise similarity (98.45%) to *D. oxyrinchus* than to *D. batis* (96.39%). These new sequences will complement available mitogenomes of batoid species, furthering investigation of population genetic differences and diversity, taxon identification, and phylogenetics of Rajiformes, thereby supporting their conservation.

**Figure 1.**
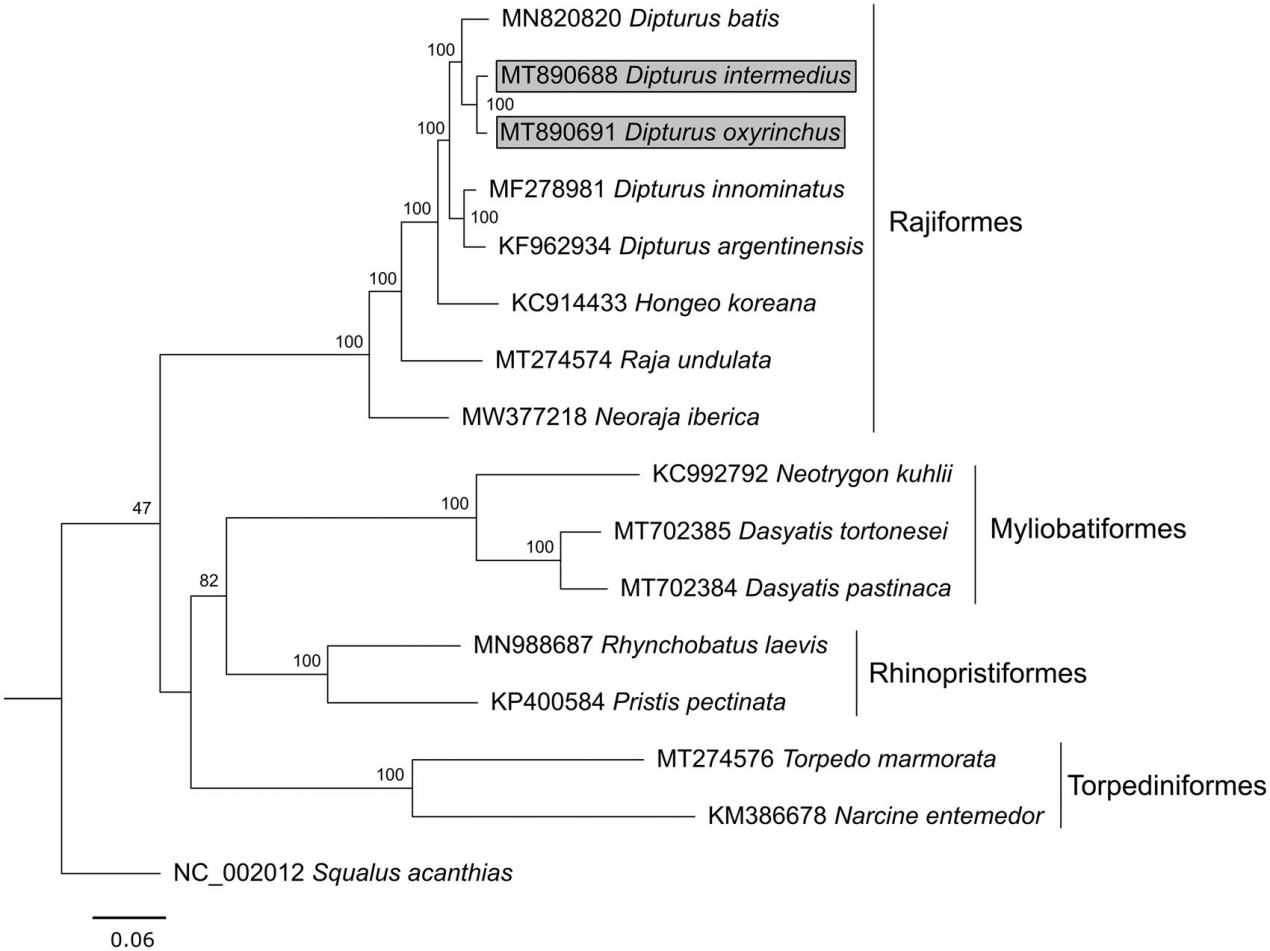
Phylogenetic tree of *Dipturus intermedius* and *D. oxyrinchus* (highlighted with gray background), 13 other batoid species and the spiny dogfish *Squalus acanthias* as outgroup, inferred from complete mitogenomes. Scale bar shows the number of substitutions, node labels display bootstrap support in percent.

## Authors contributions

Tanja N. Schwanck, Aurelien N. Delaval, Leslie R. Noble, Peter J. Wright, David W. Donnan, and Catherine S. Jones contributed to the study conception and design. Primer design, molecular work and analysis were performed by Tanja N. Schwanck under the supervision of Catherine S. Jones. Findings were interpreted and discussed among Tanja N. Schwanck, Aurelien N. Delaval, Leslie R. Noble, Peter J. Wright, David W. Donnan, and Catherine S. Jones. The first draft of the manuscript was written by Tanja N. Schwanck. Aurelien N. Delaval, Leslie R. Noble, Peter J. Wright, David W. Donnan, and Catherine S. Jones commented on previous versions of the manuscript, and together with Tanja N. Schwanck approved the final manuscript and agree to be accountable for all aspects of the work.

## Data Availability

The genome sequence data that support the findings of this study are openly available in GenBank of NCBI [https://www.ncbi.nlm.nih.gov] under the accession numbers MT890688 and MT890691; https://www.ncbi.nlm.nih.gov/nuccore/MT890688.1/; https://www.ncbi.nlm.nih.gov/nuccore/MT890691.1/
